# Comparing Spider Sampling Methods in a Eucalypt Forest in Wet and Dry Conditions

**DOI:** 10.3390/ani16101481

**Published:** 2026-05-12

**Authors:** Rachael Harris, Robert Raven, Andrew Maxwell, Peter J. Murray

**Affiliations:** School of Science, Engineering & Digital Technologies, University of Southern Queensland, Toowoomba 4350, Australiaandrew.maxwell@unisq.edu.au (A.M.); peter.murray@unisq.edu.au (P.J.M.)

**Keywords:** comparison, diversity index, species richness, spider, survey, vibration, rainfall

## Abstract

Environmental variability, such as fluctuations in rainfall, can strongly influence spider population dynamics and assemblage composition. The study examined whether rainfall in the preceding three months, i.e., wet or dry conditions, using three different methods in the same sites within a eucalyptus forest, influenced the overall species and abundance of spiders captured and thus how wet or dry conditions influenced the overall spider assemblage and community structure. In south-east Queensland, Australia, rainfall is highly variable throughout the year and does not conform to distinct wet or dry seasons; therefore, “wet” and “dry” classifications in this study refer specifically to preceding rainfall conditions rather than seasonal categories. Sampling was conducted during dry conditions (75 mL of rain in the three months preceding collection) and during wet conditions (300 mL of rain in the three months preceding collection) to assess overall differences in spider richness, diversity and assemblage composition between wet and dry conditions by combining data from the three sampling methods. Species richness was significantly higher in dry conditions compared to the wet conditions. Diversity indices indicated that the more common spider species remained consistent between wet and dry conditions. Ordination analyses revealed changes between wet and dry conditions, primarily driven by fluctuations with less common spider species, rather than community changes at the family level. For vibration-based collections of spiders, only 30.5% of species overlapped between wet and dry conditions, whereas family-level overlap was 75%, indicating rainfall-driven changes occurred primarily at the species level rather than at the family level. Spider abundance was consistently higher under dry conditions across all methods. In vibration-based collections, 90% of species was collected within 60 min. However, using this method, species appeared more slowly under wet conditions, suggesting that rainfall may influence spider responses to vibrational stimuli. Survey method strongly influenced species richness with night hand collection of spiders resulting in the greatest number of species observed. Spider species richness and diversity collected using the vibration-based method were similar in wet and dry conditions and between sites. This supports the reliability of this method for sampling spiders that respond to vibrations and should be used complementarily with other survey methods.

## Simple Summary

Spiders have an important role as a generalist predator and prey in many ecosystems and are commonly used in environmental monitoring studies due to their sensitivity to subtle environmental changes including rainfall. This study examined how wet and dry conditions affect spider communities in the same location, in open forest, using the following three spider collection methods: nocturnal hand collection, pitfall traps, and ground vibration-based method. More spider species were collected during dry than wet conditions, although the most abundant common species remained consistent. Changes in the spider community were mostly due to rare species rather than changes in spider families. For the vibration-based method, a third of the spider species collected were found in both wet and dry conditions; however, two-thirds of the spider families collected were found in both wet and dry conditions, indicating rainfall affects species-level differences for spider populations. The abundance of spiders was greater in dry conditions and their response to vibration-based spider collection method differed depending on rainfall. These findings indicate that changes in environmental conditions such as rainfall may influence spider species populations and community compositions.

## 1. Introduction

Spiders are among the most common, diverse, and abundant generalist predators in terrestrial ecosystems, playing critical roles in maintaining ecological balance [1,2,3]. Spider species vary in diel activity, occupy a range of vegetative strata, and differ widely in mobility [4,5,6]. As spiders are highly sensitive to any change in habitat structure, disturbance, and environmental conditions such as vegetation complexity, litter depth or microclimate characteristics (temperature, moisture, wind and light), they are often widely used as bioindicators of ecosystem health [3,6,7]. Alterations to spider assemblages can serve as an indicator of an ecological impact occurring at lower trophic levels [3,8]. However, spiders can be challenging to sample due to their diverse behaviours and use of microhabitats. Accurately characterising spider assemblages and community composition is essential for ecological research and conservation, as different survey methods often yield different estimates of species richness, abundance or the representation of functional guilds [5,6,9,10]. For many spider species, rainfall strongly influences activity patterns, population abundance, and prey availability [8]. However, increases in spider abundance may not occur immediately following rainfall events. As spider populations depend on many factors that influence prey availability and spider reproductive maturity, there is often a temporal lag between rainfall events, increases in vegetation productivity, increases in prey populations, and resulting increases in overall spider populations [8,11,12]. As no single survey method captures this diversity of spiders, methodological comparisons are vital to understanding biases and improving community assessments [13,14,15,16]. As spiders are crucial to ecosystems, it is important to understand how rainfall affects the ability of different survey methods to estimate spider diversity and abundance.

Traditional survey methods such as hand collection and pitfall trapping remain widely used when surveying spiders [16,17,18]. Nocturnal hand collection is effective for detecting visually active hunters but is strongly dependent on observer effort and the detectability of individual spiders [16,17,19,20]. In contrast, pitfall traps provide an efficient way of capturing mobile and ground-dwelling taxa but tend to under-sample less active or arboreal spider species [9,18,21,22,23]. Recently, vibration-based sampling was developed as a novel approach to surveying spiders, targeting spider species that may not be captured with the other two more conventional methods [6]. However, the consistency and repeatability of vibration-based sampling relative to the two traditional methods remain untested.

Environmental variability can influence ecological communities and the outcomes of biodiversity surveys to assess those communities [8,24]. Among climatic factors, rainfall is an important factor and affects various ecological processes in terrestrial ecosystems [8,25]. For spiders, rainfall affects abundance, activity, and detectability by altering prey availability, predator activity, vegetation growth, soil moisture and microclimatic conditions [26,27,28]. Invertebrate populations, the primary food source for many spiders, often fluctuate in response to climatic conditions [28]. Wet conditions typically promote plant growth and increased vegetation cover, which in turn provide more structural complexity (e.g., leaf litter, foliage) and more foraging and breeding sites for insects [8,27,29]. In turn, spiders can benefit from higher prey availability, resulting in a greater abundance and species richness during wet conditions [28,30]. Conversely, dry conditions can lead to reductions in vegetation cover resulting in simpler habitats that may not support the same diversity of spiders, and a reduction in insect populations leading to a decrease in food availability for spiders [8,28,30,31]. Variation in prey abundance or availability can directly influence spider behaviour, reproductive success and survival rates [29,32]. Additionally, other factors such as humidity and temperature, influenced by rainfall, can affect spider activity levels [33,34]. Spiders’ responses to prolonged rainfall can provide insights into the broader ecological impacts of climatic fluctuations [29]. Comparing the outcomes of spider surveys in the same sites conducted after prolonged dry and wet conditions provides an opportunity to assess how rainfall interacts with sampling methods and spider communities. These comparisons can reveal how environmental factors can influence the detectability and representation of spider species using different survey methods.

While traditional survey methods such as hand collection and pitfall trapping have been well-studied and documented in all different climates and conditions, there has been no assessment of vibration-based surveying, in relation to its repeatability and reliability in dry and wet environmental conditions [6,16,17,18]. Vibration-based collection relies on the transmission of soil substrate-borne vibration, which can be influenced by substrate composition and its moisture content. If vibration propagation characteristics affect spider responsiveness to the stimuli, environmental conditions such as rainfall may alter the effectiveness of vibration-based collections. To address this gap, this study compares three survey methods—pitfall trapping, nocturnal hand collection, and the vibration-based method, conducted in dry and wet conditions in the same sites within open forest. Thus, the aims are to (1) compare spider species richness and diversity determined using the three methods, (2) compare whether comparatively wet and dry conditions influence spider abundance and richness using these three sampling methods between the two survey rounds, (3) evaluate species accumulation patterns during vibration sampling at 10 min intervals and (4) assess the reliability of the vibration-based method as a complementary tool for spider surveys. Understanding the effects of prolonged rainfall on spider assemblages is important for successful management of biodiversity and conservation.

## 2. Materials and Methods

### 2.1. Study Area

This study was conducted within the Karrawatha–Flinders Corridor, at Stewartdale, a 1200 ha property located 46 km south-west of Brisbane, in Queensland, Australia. The Karrawatha–Flinders Corridor spans 60 km of open eucalypt forest, and Stewartdale is adjacent to the White Rock Conservation Area in White Rock, Queensland, Australia. The study sites within open forest at Stewartdale is dominated by ironbark (*Eucalyptus sideroxylon*), grey gum (*E. punctata*), and blackbutt (*E. pilularis*) [35]. Stewartdale contains both regrowth and remnant dry sclerophyll forest, while open areas were primarily covered by the grass species *Setaria sphacelate* and *Chloris gayana* [35].

### 2.2. Sampling Methods

There were two rounds of spider surveys that occurred across 14 months. The first round of surveying for spiders using pitfall traps and night collection was conducted in spring from 2 September to 1 October 2020 and the second round occurred in summer from 13 January to 25 February 2021 [6]. The first round using the vibration-based spider collection method was conducted in spring on 15–16 October 2020 and the second round occurred in spring on 4–5 November 2021. There were delays in the second round of collection due to COVID-19 lockdowns, seasonality and availability of access to the Stewartdale property. In the three months prior to the first round of spider collections (June, July and August 2020), there was a total of 75.2 mm of rain, whilst for the three months prior to the second round (November, December 2020 and January 2021) there was a total of 300.2 mm of rainfall [36,37]. The total rainfall during the first round was 5.4 mm and during the second round it was 99 mm [36,37]. The total rainfall for the three months prior to using the vibration-based spider collection method in November (August, September, and October 2021) was 194.8 mm [37]. The mean minimum to mean maximum monthly temperatures for September in 2020 were 10.3 °C to 27.5 °C and in October 13.0 °C to 29.7 °C for the first round of spider collections [38,39]. The mean monthly temperatures for January were 19.2 °C to 30.4 °C, 19.2 °C to 31.5 °C in February and in November 17.2 °C to 27.8 °C for the second round of spider collections [40,41,42].

**Figure 1:**
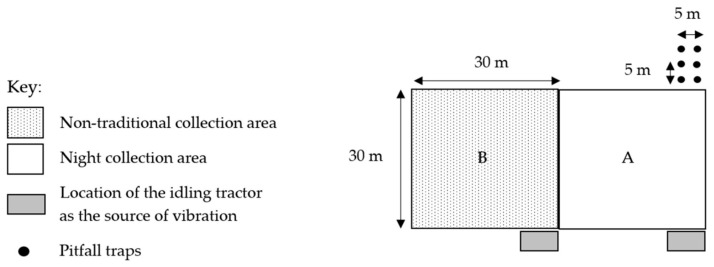
Layout of the survey methods used to collect spiders with sites A and B in four similar locations within eucalyptus forest on the property of Stewartdale in southeast Queensland.

In open dry sclerophyll woodland, four vegetatively similar 30 × 30 m (900 m^2^) sites were marked with white reflective tape at each corner. These four sites had an adjacent 30 × 30 m area and were labelled site A and site B (Figure 1). Site A was used for a combination of the night hand collection of spiders, pitfall traps and vibration-based spider collection method (Figure 1). Site B was used for the vibration-based spider collection method to test if the vibration-based spider collection method was impacted by the night collection of spiders and pitfall trapping. Each of these four sites were labelled DR1, DR2, RL, and RH. An ethical exemption to collect spiders was approved by the University of Southern Queensland Animal Ethics Committee (exemption ID 20EXE004).

### 2.3. Nocturnal Hand Collection

The night collection was conducted within the bounds of the 900 m^2^ site for one hour once a fortnight for three consecutive fortnights for each round of spider collections. The nocturnal hand collection at each site was split into two 30 min intervals whereby two people collected spiders found above 0.5 m while another person focused on collecting spiders from vegetation below knee height and within the leaf litter on the ground. After 30 min, the roles were exchanged and the collection of spiders continued for another 30 min. At each of the four sites spiders were collected into 50 mL yellow screw cap labelled specimen containers containing 70% ethanol. This procedure was followed in rounds 1 and 2.

### 2.4. Pitfall Trap Collection

At each of the four sites, six plastic pitfall traps (600 mL, 6 cm diameter) containing 100 mL of propylene glycol were installed outside the 900 m^2^ site (Figure 1). The traps were positioned 5 m apart in two parallel rows beginning at the back corner of each site. Each pitfall trap had a shelter placed above it to prevent entry or minimise disruption to the pitfall trap by rain, reptiles, amphibians, or small mammals. These shelters were made from a face-down plastic plate with three wooden skewers placed evenly apart around the perimeter of the plate. The spiders captured in the pitfall traps were collected every fortnight on the day of the nocturnal spider collections, with a total of 1008 trap nights for each round. Each pitfall trap was emptied into another 600 mL container, and the pitfall trap was reset with the lid and shelter both back in place.

### 2.5. Vibration-Based Collection

The vibration-based spider collection method used a John Deere tractor (model 6520SE idling at 750–800 rpm, Deere and Company, Moline, IL, USA) as the vibration source at each of the four sites after the pitfall trapping and the nocturnal hand collections were completed. Vegetation was cleared in an area large enough to include the tractor with a 1 m bare strip around the tractor to ensure visibility of any spiders attracted to the vibration. Between midday and dusk, the tractor was parked in the cleared area with the engine left to idle and spiders were collected for 60 min (as six 10 min collections) at each collection site. Three people were stationed at the front, middle and rear of the right side of the tractor for collections. Spiders were only captured if they were moving towards the tractor in the cleared space. Spiders were collected in a 50 mL yellow screw cap container containing 70% ethanol. This was repeated for each of the four sites for rounds 1 and 2.

### 2.6. Identification

The contents of each collection container containing spiders were poured into a 100 mm Petri dish with 70% ethanol, the Petri dish was then placed under a Nikon dissection microscope (Nikon Corporation, Tokyo, Japan), and spiders were identified using 10× magnification. Whether the spider was male, female, or too juvenile to determine, its gender was recorded. Photographs were taken of the dorsal and ventral sides of each spider for later reference. For the pitfall trap spider collection, spiders were kept in separate containers for each site and labelled accordingly. These processes were repeated for each site and for rounds 1 and 2. Spiders of all instars were identified and checked by Dr Robert Raven, who has over 40 years of experience as a professional arachnologist. Young spiders of different ages were linked by a sequence from very young to adult. A placeholder name was used for species that could not be identified at that time in the format of the first three letters of the genus followed by a number that represented individual species, e.g., *Habronestes* sp. 1 was written as *Habronestes* hab1. This designation does not denote or suggest a new species unknown to science.

### 2.7. Statistical Analyses

The data were analysed using a two-way ANOVA with model terms for the site, the condition (wet or dry), and the survey methods to determine the Shannon’s diversity index, Simpsons diversity index and species richness. This was completed using R (version 4.0.5), with the R packages “readxl”, “ggplot”, “emmeans”, “multcomp”, “vegan”, and “tidyverse”. The analyses compared the following three survey methods: night collection, pitfall traps, and vibration-based spider collection conducted at site A for both wet and dry conditions (Table 1). Vibration sites A and B were also compared in both wet and dry conditions (Table 2). Significance was expressed in different superscript groupings (“a”, “b” and/or “c”) with a pooled SEM and confidence level under each Table.

## 3. Results

The total number of spiders collected at Stewartdale (N = 4383) across the two rounds, i.e., collection periods, were identified into a total of 35 families, 151 genera, and 268 species (the complete list of spider species is given in Appendix A). A total of 2413 spiders were captured in night collection, 539 spiders in pitfall traps, and 1431 spiders were captured in the vibration-based method. Spider species richness differed significantly between wet and dry conditions (F1,15 = 11.144, *p* = 0.0045). In contrast, spider species diversity did not differ between wet and dry conditions when measured using the Shannon’s diversity index (F1,24 = 2.45, *p* = 0.13), and the Simpsons index (F1,21 = 0.28, *p* = 0.60) (Table 1).

The data from the two vibration-based collection sites at each location were analysed to assess the species richness and diversity (using Shannon’s diversity index and Simpsons diversity index) between wet and dry conditions. Species diversity did not differ between wet and dry conditions when measured using Shannon’s diversity index (F1,13 = 0.07, *p* = 0.80) or Simpsons diversity index (F1,13 = 0.001, *p* = 0.997). Similarly, the species richness did not differ between wet and dry conditions (F1,13 = 015, *p* = 0.70) or between collection sites A and B (F1,13 = 1.77, *p* = 0.21) for the vibration-based collection method (Table 2).

**Figure 2:**
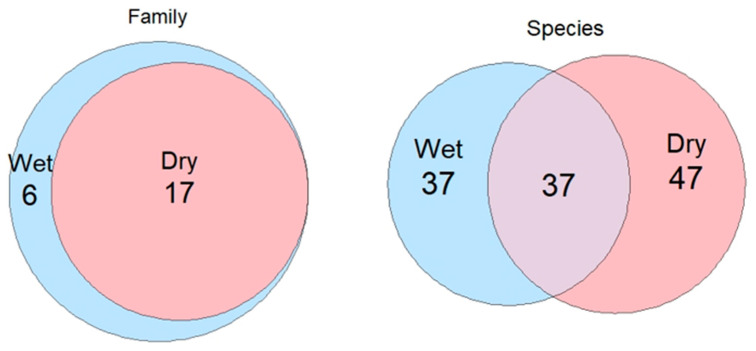
Proportionally accurate Venn diagram of unique spider families (**left**) and species (**right**) captured in wet compared to dry conditions using the vibration-based collection method at sites A and B within eucalyptus forest on the property Stewartdale in southeast Queensland.

Combining data from sites A and B, for the vibration-based spider collection method, confirmed that the spider species composition varied between wet and dry conditions, with more unique spider species collected using this method in dry than wet conditions. Furthermore, only 30.5% of the spider species captured using the vibration-based spider collection method were captured in both wet and dry conditions. In contrast, the number of unique families found was higher in wet conditions than in dry conditions, with 65.3% of families captured in both wet and dry conditions. Spider family composition showed six families unique to wet conditions, with all families recorded in dry conditions also present in wet. In contrast, species-level patterns differed with 10 additional unique species recorded under dry conditions compared to wet conditions (Figure 2).

**Figure 3:**
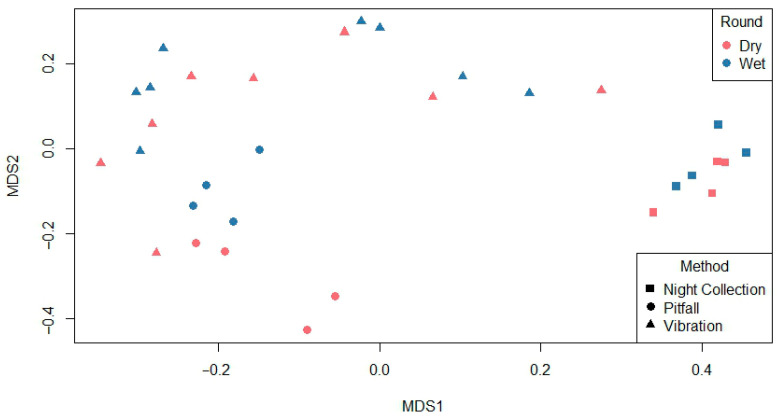
Bray–Curtis non-metric multidimensional scaling (NMDS) ordination showing dissimilarity of spider family assemblages between wet and dry conditions for the three spider collection methods used in eucalyptus forest on the property Stewartdale in southeast Queensland.

Night collection samples form a distinct cluster on the right, while pitfall trap and vibration-based method samples are more dispersed and partially overlap. There was some similarity of spider family assemblages between wet and dry conditions for the three spider collection methods (Figure 3).

**Figure 4:**
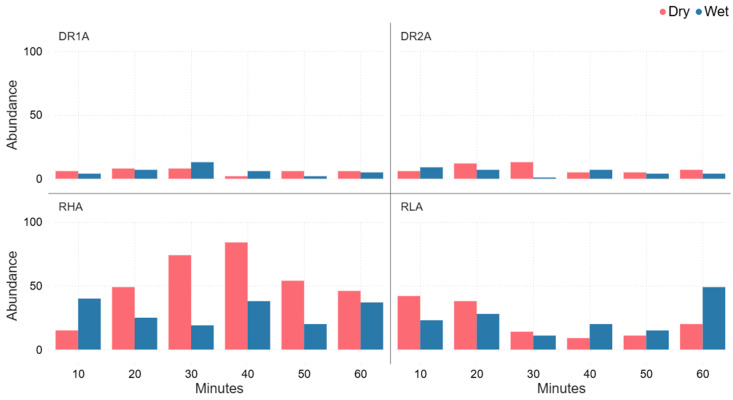
Comparison of the spider abundance for the four vibration-based spider collection A sites, i.e., DR1, DR2, RH, and RL, for each 10 min continuous sampling time for a total of 60 min, for spiders collected under wet and dry conditions within eucalyptus forest on the property Stewartdale in southeast Queensland.

Spider abundance varied by site and time with RHA’s consistently higher abundances, particularly under dry conditions, while DR1A and DR2A remained relatively low across all time collection periods. Differences between wet and dry conditions were site-dependent, with dry conditions generally higher at the start of the time collection period, and with wet conditions having a higher abundance towards the end of the time collection period (Figure 4).

**Figure 5:**
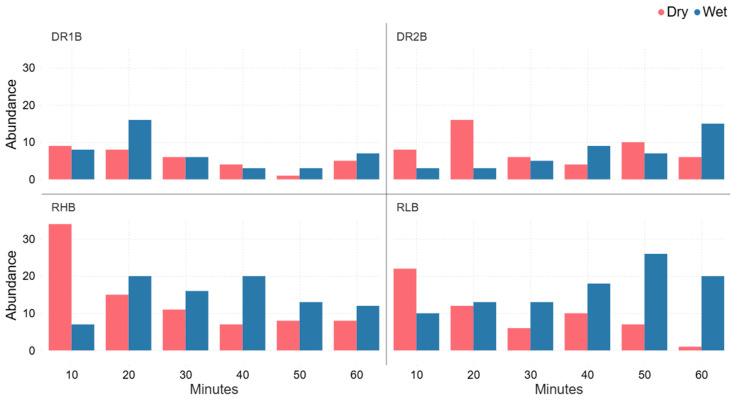
Comparison of the spider abundance for the four vibration-based spider collection B sites, i.e., DR1, DR2, RH, and RL, for each 10 min continuous sampling time for a total of 60 min, for spiders collected under wet and dry conditions within eucalyptus forest on the property Stewartdale in southeast Queensland.

Spider abundance varied by site and time, with RHB showing a strong peak under dry conditions within the first 10 min of collection, while wet conditions were generally higher towards the end of the time collection period. DR1B and DR2B remained relatively low, whereas RLB showed a clear increase in abundance under wet conditions over time, particularly at 50–60 min (Figure 5).

**Figure 6:**
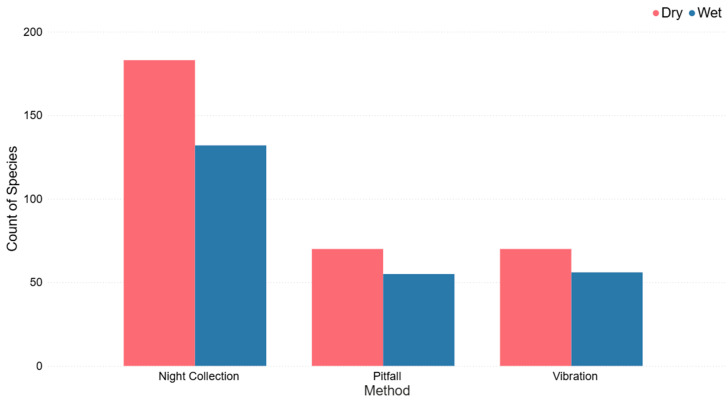
Spider species richness for the following three methods: night collection, vibration-based (site A), and pitfall traps for spiders collected under both wet and dry conditions within eucalyptus forest on the property Stewartdale in southeast Queensland.

Night collection had the highest species richness in both wet and dry conditions, with a very similar species richness in pitfall traps and vibration-based collection for both wet and dry conditions (Figure 6).

**Figure 7:**
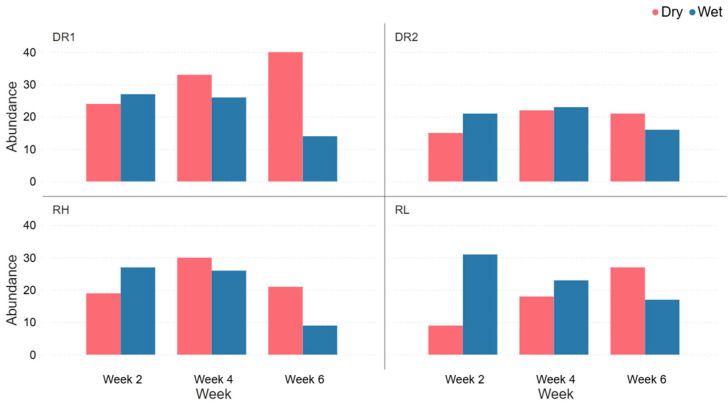
Spider abundance in pitfall trapping over 6 weeks for spiders collected under wet and dry conditions at each site within eucalyptus forest on the property Stewartdale in southeast Queensland.

Spider abundance varied by site and time, with a general trend across sites of a higher spider abundance for wet conditions at the start of the collection period and lower at the end, and conversely, lower spider abundance for dry conditions at the start of the collection period and higher at the end (Figure 7).

**Figure 8:**
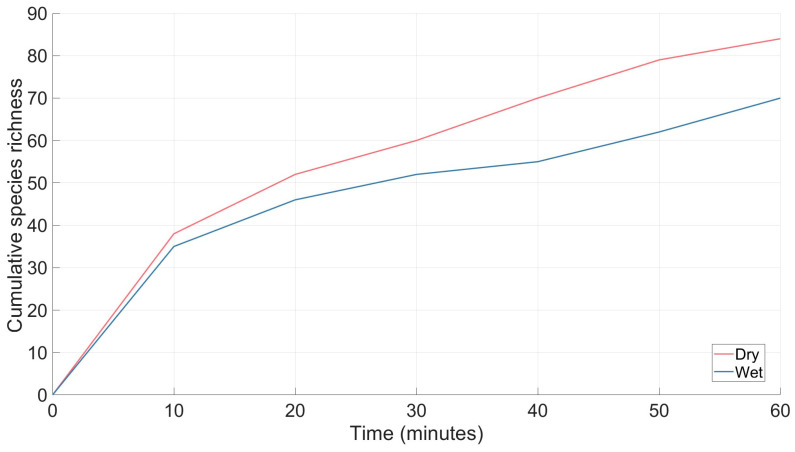
Species accumulation curve over 60 min for spiders collected under wet and dry conditions for vibration-based collections within eucalyptus forest on the property Stewartdale in southeast Queensland.

In dry conditions, there was a greater species richness than in wet conditions (Figure 8). In wet and dry conditions, species richness had not reached a plateau by the 60 min collection period (Figure 8).

**Figure 9:**
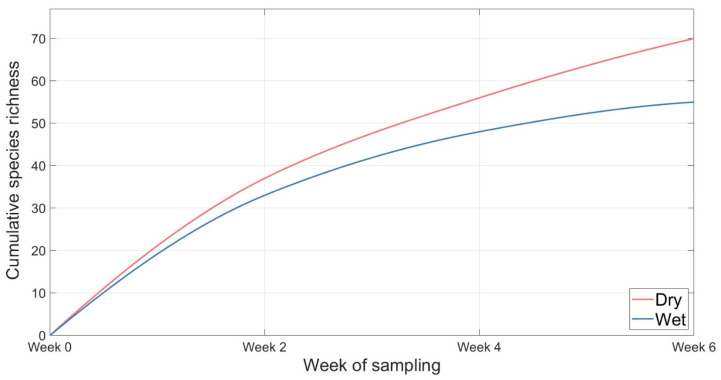
Species accumulation curve over 6 weeks for spiders collected in pitfall traps under wet and dry conditions within eucalyptus forest on the property Stewartdale in southeast Queensland.

In dry conditions there was a greater species richness than in wet conditions. In dry conditions there was a steady increase in spider species richness across the six weeks with no clear plateau, while wet conditions slowed after four weeks, starting to plateau from week 6 (Figure 9).

## 4. Discussion

This study examined how rainfall in the months preceding collection creating either a comparatively wet condition or dry condition, using a vibration-based collection method, night hand collection of spiders and pitfall traps, influences overall spider assemblage and community composition. Species richness was significantly higher in dry conditions compared to wet conditions. The Shannon’s species diversity index was significantly different between wet and dry conditions for night collection and pitfall traps, but not for the vibration-based method; however, the Simpsons species diversity index was not significantly different between wet and dry conditions across all three methods (Table 1). This indicates that changes in species richness were driven mainly by rare species/singletons while the dominant taxa remained relatively consistent. Rainfall influences vegetation structure, microclimatic conditions and prey availability, all of which may selectively affect less abundant species with a narrow ecological niche [8,27]. Conversely, our findings also indicate a degree of resilience in the core spider assemblage relative to short-term environmental variability, consistent with previous Australian studies [8,30].

Spider community composition varied between wet and dry conditions. Ordination analyses revealed separation of assemblages between wet and dry conditions (Figure 3). While there was some overlap between wet and dry conditions, wet conditions tended to cluster slightly apart from the collections of spiders in dry conditions, indicating temporal variation in species composition. In contrast, sampling method showed substantial overlap within the ordination space, with no clear separation between methods suggesting strong site-level family composition. Species overlap analyses indicated species turnover within vibration-based collections (Figure 2). Only 30.5% of species were recorded in both wet and dry conditions using the vibration-based method, whereas the family composition remained comparatively stable with 75% overlap between conditions. No spider families were exclusive to dry conditions, with six families exclusively found in wet conditions (Figure 2). This suggests that rainfall in the months preceding collection may influence the species level rather than the family level, reflecting shifts in species-specific activity patterns. Spiller and Schoener [26] and Langlands, Brennan and Pearson [8] had comparable patterns of seasonal turnover, where the amount of rainfall has altered species presence without changing the overall community structure.

Spider species richness and diversity estimates differed among survey methods. Nocturnal hand collection of spiders consistently resulted in the highest species richness in both wet and dry conditions, possibly due to the presence of known visually active foliage-dwelling spiders and increased sampling time and effort [6,17]. In contrast spiders collected in pitfall traps and by the vibration-based collection method had lower species richness but comparable species diversity indices, suggesting these methods sample a narrower section of this spider community (Table 1). Pitfall traps have biases towards capturing mobile, ground-active spider species whilst underestimating arboreal or less-active taxa, particularly under varying environmental conditions [18,22]. Vibration-based collection of spiders also has a bias towards species that are behaviourally reactive to the vibration [6]. However, across all methods, spider species richness was higher in dry than in wet conditions (Figure 6). Although prolonged wet conditions can increase prey availability and vegetation structural complexity (leaf litter or foliage) which can in turn be beneficial for spider abundance, wet periods can also increase shelter availability and reduce the need for spiders to move with lower foraging activity due to factors such as wind, saturation and humidity [8,27,29]. Conversely, dry conditions can also promote reduced shelter availability, increase foraging movement, and increase ground surface activity which in turn can increase the probability of capturing spiders [8,28,30,31]. Whilst there were significant species-level differences between collection methods, there was considerable overlap between methods at the spider family level (Figure 3).

The vibration-based spider collection method demonstrated consistency between wet and dry conditions and between sampling sites (Table 2). Species richness and diversity of spiders did not differ significantly between wet and dry conditions, nor between sites A and B, indicating the method is relatively robust to relatively short-term environmental variability. This suggests that vibration-based collections of spiders provide repeatable estimates of spider diversity for the species responsive to vibrational stimuli supporting its reliability as a complementary survey method.

During the 60 min collection of spiders at the vibration-based spider collection sites A and B, spider abundance was higher at sites RL and RH than at sites DR1 and DR2 (Figure 4 and Figure 5). This may have been influenced by site topography as RL and RH spider collection locations were located at the base of a slope, whereas DR1 and DR2 spider collection locations were situated on slope crests. Slope position may affect spider movement or energetic costs associated with responding to vibrational stimuli potentially influencing the likelihood of individuals reaching the vibration source. Although further research is required to confirm this mechanism, this observed pattern suggests that small-scale habitat features may influence spider responsiveness during vibration-based spider surveys. Figure 4, Figure 5 and Figure 7 demonstrate that even visually similar open forest sites can differ in spider richness and abundance. These results indicate that fine-scale environmental factors, such as slope, may influence spiders’ community composition. As a result, studies conducted on a single site may underestimate the diversity and abundance of spiders in that location. Comparatively, spider abundance was higher in wet conditions in week 2 and declined over the following four weeks, whereas abundance in dry conditions increased from week 2 and peaked in week 6 (Figure 7). This pattern suggests that wet/dry conditions influence spider activity over time, potentially affecting movement behaviour and capture rates in pitfall traps.

The spider species accumulation curves for dry conditions indicated that the vibration-responsive species were detected early in the sampling period in the first 20 min of sampling (Figure 8). This was followed by a progressively slower rate of accumulation between 20 and 40 min. By 60 min, the curve has begun to plateau with only a small increase (five new species) in the last 10 min of sampling, indicating even less accumulation past the 60 min mark. Approximately 90% of species observed were collected within 50 min of sampling (Figure 8). In contrast, the wet condition spider species accumulation curve increased rapidly in the first 20 min of sampling, increased by three additional species between 30 and 40 min, and then had 12 new species in the last 10 min sampling period between 50 and 60 min, suggesting sampling had not yet reached a plateau (Figure 8). This could suggest a delayed response by spiders to vibrational stimuli in wet conditions. The propagation speed of vibration waves across soil are highly dependent on substrate properties and moisture levels. For example, Aicher and Tautz [43] demonstrated that surface waves from vibration travelled faster in wet sand than in dry sand. Furthermore, a study by Wu et al. [44] indicated that spiders may not respond to vibration waves exceeding approximately 60 m s^−1^, whereas responses were observed when vibration wave speeds were below 60 m s^−1^ within frequencies between 20 and 200 Hz. This is thought to be because the wave length at lower speeds is more comparable to the size of the spider facilitating mechanoreception detection [44]. Variation in substrate moisture between wet and dry conditions may therefore influence vibrational transmission properties, potentially altering spider responsiveness to the vibrations from the tractor contributing to a delayed accumulation of spider species during surveys in wet conditions.

Pitfall trap spider species accumulation curves showed continued increases in species richness over time in both dry and wet conditions (Figure 9). In dry conditions, spider species richness increased steadily, across the six weeks with no clear plateau. In contrast, spider species accumulation in wet conditions slowed after four weeks, potentially indicating less accumulation beyond 6 weeks of sampling. Differences in accumulation patterns of wet and dry conditions may reflect conditional differences in activity with a greater number of ground-active spiders in dry conditions than in wet conditions. These results suggest trapping duration influences sampling completeness across environmental conditions and indicates a longer deployment period may be required for sampling completeness using pitfall traps. While we have characterised the two rounds presented comparatively as a wet condition or dry condition, rainfall alone is unlikely to be the sole driver of observed variation in spider abundance, diversity, and assemblage composition. Other uncontrolled variables may have contributed to the patterns observed as spider activity, life stage and distribution, and community structures are known to fluctuate with seasonal cycles and environmental conditions.

## 5. Conclusions

In this study rainfall influenced spider assemblage primarily at the species level rather than the family level, with a higher species richness recorded during dry conditions, whilst family composition remained relatively stable between wet and dry conditions. Differences between wet and dry conditions were mainly driven by changes in less common species, whereas the most common species remained relatively stable. This indicates resilience among the core (more common) spider assemblage but sensitivity from less abundant species. Sampling method also significantly influenced estimates of spider density, with night collection having the greatest species richness. Despite this, the vibration-based spider collection method had consistency in species richness between sites A and B and between wet and dry conditions, supporting the methods’ reliability for detecting spider species responsive to vibration-based sampling. Accumulation curves showed that sampling duration requirements may differ slightly between wet and dry conditions, but that 60 min using vibration is sufficient for collecting 90% of spider species collected using this method. Reduced spider abundance captured from the vibration-based spider collection method in wet conditions compared to dry may reflect moisture-induced changes in vibration transmission, which could either delay spider responses or decrease reactivity to the vibrational stimuli. The sampling duration needed for pitfall traps needs to be longer than 6 weeks; there was a reduced abundance of spiders in wet than in dry conditions. These results show how environmental conditions and survey method used can shape the observed pattern of spider richness, diversity and assemblage composition. Implementing multiple survey methods whilst also accounting for environmental conditions should provide a comprehensive understanding of spider community dynamics and can improve the reliability of biodiversity surveys. As the vibration-based spider collection method is a relatively new survey method, further research is needed to better define its scope and limitations and underlying mechanisms. It is recommended that the ground vibrations the tractor produces be characterised to further understand the mechanisms driving spider responses to vibration-based collections. Additionally, the influence of environmental variable such as temperature and soil moisture on spider responses to vibration-based sampling should also be investigated. Advancing understanding of vibrational wave transmission and spider behavioural responses will strengthen the reliability of the vibration-based spider collection method and help clarify the conditions under which this method is most effective and improve its application in ecological surveys.

## Figures and Tables

**Table 1:** Comparison of mean values for spider species richness and diversity indexes for the three survey methods used in wet and dry conditions, within eucalyptus forest on the property Stewartdale in southeast Queensland.

Method	Condition	Shannon’s Diversity Index	Simpsons Diversity Index	Species Richness	No. Species
Night collection	Dry	5.84 ^a^	0.976 ^a^	92.7 ^a^	181
	Wet	5.35 ^ab^	0.962 ^a^	70 ^b^	132
Pitfall traps	Dry	4.37 ^bc^	0.945 ^a^	30.2 ^c^	68
	Wet	4.13 ^c^	0.948 ^a^	26.8 ^c^	55
Vibration site A	Dry	3.88 ^c^	0.904 ^a^	29.2 ^c^	70
	Wet	3.65 ^c^	0.915 ^a^	24.2 ^c^	56
Pooled SEM		0.286	0.121	3.82	
Confidence level		0.95	0.95	0.95	

^a, b, c^ within each column means followed by the same superscript letter were not significantly different.

**Table 2:** Comparison of mean values for species richness and diversity indexes for the two sites where vibration-based spider collections took place in wet and dry conditions, within eucalyptus forest on the property Stewartdale in southeast Queensland.

Method	Condition	Shannon’s Diversity Index	Simpsons Diversity Index	Species Richness	No. Species
Vibration site A	Dry	3.87 ^a^	0.910 ^a^	27.6 ^a^	70
	Wet	3.83 ^a^	0.910 ^a^	25.9 ^a^	56
Vibration site B	Dry	3.82 ^a^	0.919 ^a^	21.6 ^a^	43
	Wet	3.78 ^a^	0.919 ^a^	19.9 ^a^	53
Pooled SEM		0.13	0.011	3.9	
Confidence level		0.95	0.95	0.95	

^a^ within each column means followed by the same superscript letter were not significantly different.

## Data Availability

Data available upon reasonable request from the authors.

## Abbreviations

The following abbreviations are used in this manuscript:

COVID-19	Coronavirus disease of 2019
ANOVA	Analysis of variance
NMDS	Non-metric multidimensional scaling
SEM	Standard error of the mean
RH	Rachael’s Hill
RL	Robert’s Lane
DR1	David’s Ridge 1
DR2	David’s Ridge 2

## Appendix A

**Table A1:** Complete species list of spiders collected in open forest at Stewartdale, south-east Queensland, Australia. Some of the spiders are deposited in the Queensland Museum, Queensland, Australia, and some in Leibniz Institute for the Analysis of Biodiversity Change, Hamburg, Germany. Registration numbers will be determined by Queensland Museum staff and is not yet finalised, i.e., the specimens are yet to receive registration numbers. We have assigned numbers to new species that have been described.

Family	Genus and Species
Ammoxenidae	Genus *A Sp.1*
Amurobiidae	*Dadurus dad1*
Araneidae	*Acroaspis acr1* *Anepsion peltoides* *Arachnura higginsi* *Araneus acuminatus* *Araneus albotriangularis* *Araneus ara1* *Araneus ara10* *Araneus ara11* *Araneus ara12* *Araneus ara13* *Araneus ara2* *Araneus ara3* *Araneus ara4* *Araneus ara5* *Araneus ara6* *Araneus ara7* *Araneus ara8* *Araneus ara9* *Araneus arenaceus* *Araneus cytarachnoides* *Araneus lodiculus* *Araneus lutulentus* *Argiope keyserlingi* *Argiope protensa* *Austracantha minax* *Celaenia cel1* *Cyclosa cyc1* *Cyclosa trilobata* *Cyclosa vallata* *Cyrobil darwini* *Cyrtophora exanthematica* *Cyrtophora hirta* *Dolophones dol1* *Dolophones turrigera* *Hortophora hor1* *Hortophora transmarina* *Larinia montagui* *Larinia phthisica* *Leviana dimidiatus* *Neoscona theisii* *Ordgarius monstrosus* *Phonognatha graeffi* *Phonognatha pho1* *Phonognatha wagneri* *Plebs eburnus* *Poecilopachys australasia* *Poltys pol1* *Trichonephila edulis*
Arkyidae	*Arkys ark1* *Arkys walckenaeri*
Clubionidae	*Clubiona clu1* *Clubiona clu2* *Clubiona clu3*
Corinnidae	*Battalus bat1* *Iridonyssus formicans* *Iridonyssus murrayman* *Iridonyssus kohouti* *Iridonyssus leucostaurus* *Nucastia nuc1* *Nyssus albopunctatus* *Nyssus coloripes* *Nyssus jaredwardeni* *Nyssus luteofinis* *Nyssus paradoxus* *Nyssus pseudomaculatus* *Poecilipta janthina* *Poecilipta kgari* *Poecilipta kohouti* *Poecilipta poe1*
Cycloctenidae	*Cycloctenus cyc1*
Deinopidae	*Asianopis subrufa*
Desidae	*Badumna bad1* *Badumna bad2* *Badumna bad3* *Barahna bar1* *Corasoides australis*
Dolomedidae	*Ornodolomedes orn1*
Eutichuridae	*Cheiracanthium che1* *Cheiracanthium che2* *Cheiracanthium che3*
Family 1	Genus *B sp.1*
Gnaphosidae	*Ceryerda cer1* *Eilica eil1* *Eilica eil2* *Eilica eil3* *Encoptarthria enc1* *Encoptarthria enc2* *Encoptarthria enc3* *Encoptarthria enc4* *Encoptarthria enc5* *Encoptarthria enc6* Genus *C sp.1* Genus *D sp.1* Genus *E sp.1* Genus *F sp.1* Genus *G sp.1* Genus *H sp.1* *Molycria dawson* *Molycria mol1* *Myandra mya1* *Zelotes zel1*
Hahniidae	Genus Y *sp.1* Genus Y *sp.3* *Hahniidae hah1* *Hahniidae hah2*
Hersiliidae Lamponidae	*Tamopsis tam1* *Asadipus asa1* *Centrothele cen1* *Centrothele cen2* Genus I *sp.1* *Lamponata daviesae* *Pseudolampona brookfield* *Pseudolampona pse1*
Linyphiidae	*Laetesia lae1* *Laperousea lap1* *Laperousea lap2*
Lycosidae	*Allocosa palabunda* *Anomalosa ano1* *Artoria art1* *Artoria art2* Genus J *sp.1* Genus L *sp.1* Genus M *sp.1* Genus N *sp.1* Genus O *sp.1* *Tasmanicosa godeffroyi* *Tasmanicosa tas1* *Venator spenceri* *Venatrix ven1* *Venonia micarioides*
Malkaridae	*Anarchaea ana1*
Miturgidae	*Argoctenus arg1* *Argoctenus arg2* *Elassoctenus ela1* Genus P *sp.1* *Mituliodon tarantulinus* *Miturga gilva* *Mitzoruga insularis* *Nuliodon fishburni* *Thasyraea tha1* *Tuxoctenus gloverae* *Zora zor1*
Nicodamidae	*Ambicodamus amb1*
Oonopidae	*Opopaea opo1*
Oxyopidae	*Oxyopes elegans* *Oxyopes oxy1* *Oxyopes oxy2* *Oxyopes oxy3*
Philodromidae	*Tibellus tenellus*
Pisauridae	*Perenethis per1*
Salticidae	*Bavia ludicra* *Cytaea cyt1* Genus Q *sp.1* Genus R *sp.1* Genus R *sp.2* Genus R *sp.3* Genus R *sp.4* Genus S *sp.1* Genus S *sp.2* Genus S *sp.3* Genus S *sp.5* *Holoplatys hol1* *Holoplatys hol2* *Holoplatys hol3* *Holoplatys hol4* *Holoplatys hol5* *Holoplatys planissima* *Maratus grissius* *Maratus mar1* *Maratus mar2* *Maratus mar3* *Maratus mar4* *Maratus mar5* *Maratus mar6* *Maratus mar7* *Maratus purcellae* *Menemerus bivittatus* *Myrmarachne myr1* *Neon neo1* *Opisthoncus opi1* *Opisthoncus opi2* *Opisthoncus opi3* *Opisthoncus opi4* *Opisthoncus opi5* *Prostheclina pro1* *Sandalodes bipenicillatus* *Sandalodes san1* *Sandalodes san2* *Simaetha sim1* *Zebraplatys zeb1* *Zenodorus orbiculatus* *Zenodorus zen1*
Sparassidae	*Delena cancerides* *Delena del1* *Holconia immanis* *Isopedella flavida* *Neosparassus diana* *Neosparassus salacius* *Pediana regina*
Tetragnathidae	*Leucauge decorata* *Tetragnatha tet1*
Theridiidae	*Achaearanea ach1* *Argyrodes alannae* *Argyrodes antipodiana* *Ariamnes colubrinus* *Cryptachaea veruculata* *Dipoena dip1* *Dipoena dip2* *Episinus bicornis* *Euryopis elegans* *Euryopis eur1* *Euryopis eur2* *Euryopis eur3* Genus T *sp.1* Genus U *sp.1* Genus V *sp.1* Genus V *sp.2* Genus W *sp.1* Genus Z *sp.1* *Janula bicornis* *Latrodectus hasselti* *Parasteatoda decorata* *Parasteatoda par1* *Parasteatoda par2* *Parasteatoda tepidariorum* *Parasteatoda threadstripes* *Phoroncidia pho1* *Phoroncidia pho2* *Probiscidula prb1* *Rhomphaea cometes* *Steatoda ste1* *Theridion albostriata* *Theridion pyramidale* *Theridion theridides* *Thwaitesia argentiopunctata* *Thwaitesia nigropunctata*
Thomisidae	*Boomerangia boo1* *Cymbacha cym1* *Cymbacha saucia* Genus X *sp.1* *Runcinia elongata* *Sidymella bicornis* *Sidymella sid1* *Stephanopis scabra* *Tharrhalea multopunctata* *Tmarus tma1* *Tmarus tma3* *Tmarus tma4* *Zygometis xanthogaster*
Trachelidae	*Orthobula ort1*
Trachycosmiidae	*Trachycosmus tra1*
Trochanteriidae	*Hemicloea hem1*
Uloboridae	*Miagrammopes mia1* *Miagrammopes sp1* *Philoponella congregabilis* *Philoponella phi1*
Zodariidae	*Euasteron enterprise* *Habronestes hab1* *Habronestes hab2* *Habronestes hab3* *Habronestes hab4* *Habronestes hab5* *Hetaerica scenica* *Neostorena neo1* *Notasteron lawlessi*
